# Progress in understanding the mechanisms of resistance to BCL-2 inhibitors

**DOI:** 10.1186/s40164-022-00283-0

**Published:** 2022-05-21

**Authors:** Yilan Xu, Haige Ye

**Affiliations:** Department of Hematology, The First Affiliated Hospital of Wenzhou Medical University-Zhejiang, Wenzhou, China

**Keywords:** Venetoclax, Resistance, BCL-2, Gene mutations, OXPHOS

## Abstract

Venetoclax is a new type of BH3 mimetic compound that can target the binding site in the BCL-2 protein and induce apoptosis in cancer cells by stimulating the mitochondrial apoptotic pathway. Venetoclax is especially used to treat haematological malignancies. However, with the recent expansion in the applications of venetoclax, some cases of venetoclax resistance have appeared, posing a major problem in clinical treatment. In this article, we explored several common mechanisms of venetoclax resistance. Increased expression of the antiapoptotic proteins MCL-1 and BCL-XL plays a key role in conferring cellular resistance to venetoclax. These proteins can bind to the released BIM in the context of venetoclax binding to BCL-2 and thus continue to inhibit mitochondrial apoptosis. Structural mutations in BCL-2 family proteins caused by genetic instability lead to decreased affinity for venetoclax and inhibit the intrinsic apoptosis pathway. Mutation or deletion of the BAX gene renders the BAX protein unable to anchor to the outer mitochondrial membrane to form pores. In addition to changes in BCL-2 family genes, mutations in other oncogenes can also confer resistance to apoptosis induced by venetoclax. TP53 mutations and the expansion of FLT3-ITD promote the expression of antiapoptotic proteins MCL-1 and BCL-XL through multiple signalling pathways, and interfere with venetoclax-mediated apoptosis processes depending on their affinity for BH3-only proteins. Finally, the level of mitochondrial oxidative phosphorylation in venetoclax-resistant leukaemia stem cells is highly abnormal. Not only the metabolic pathways but also the levels of important metabolic components are changed, and all of these alterations antagonize the venetoclax-mediated inhibition of energy metabolism and promote the survival and proliferation of leukaemia stem cells. In addition, venetoclax can change mitochondrial morphology independent of the BCL-2 protein family, leading to mitochondrial dysfunction. However, mitochondria resistant to venetoclax antagonize this effect, forming tighter mitochondrial cristae, which provide more energy for cell survival.

## Introduction

**Fig. 1:**
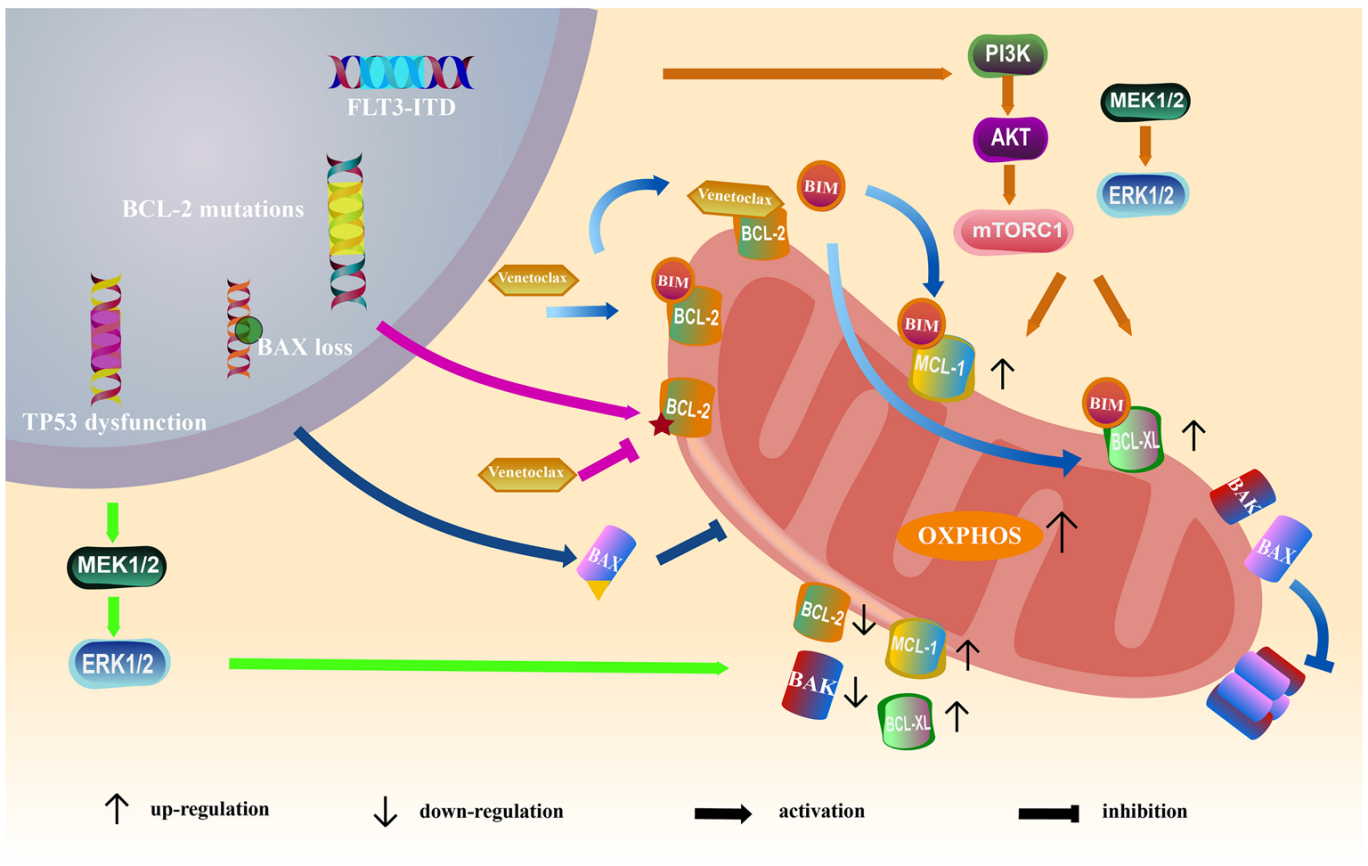
The mechanisms of resistance to BCL-2 inhibitors

The phenomenon of apoptosis resistance is an important indicator of the occurrence and development of haematological malignancies. Blocking apoptosis causes cancer cells to proliferate uncontrollably [1, 2]. BCL-2 family proteins play an important role in the mitochondria-mediated intrinsic apoptosis pathway. These proteins are a class of proteins with similar domains and are divided into 3 main categories: antiapoptotic proteins, proapoptotic proteins and regulatory proteins. The regulatory proteins contain only the BH3 domain and are thus called BH3-only proteins. If a BH3-only protein binds to the antiapoptotic protein BCL-2, BCL-2 can no longer bind to the proapoptotic proteins BAX/BAK, inhibiting their recruitment and thereby blocking the proapoptotic pathway. However, binding of a BH3-only protein to BAX/BAK can promote the recruitment and oligomerization of BAX/BAK, thereby resulting in the formation of pores on the outer mitochondrial membrane, which release cytochrome C, leading to proteolysis and apoptosis [3, 4]. Therefore, apoptosis and survival are balanced by regulating antiapoptotic proteins and proapoptotic proteins. Abnormal expression of BCL-2 family proteins is a common finding in haematological malignancies, the most important of which is overexpression of BCL-2. High levels of BCL-2 can be observed in patients with follicular lymphoma (FL), chronic lymphocytic leukaemia (CLL), mantle cell lymphoma (MCL) and Waldenström's macroglobulinaemia [5, 6]. Therefore, in BCL-2-dependent haematological malignancies, BCL-2 inhibitors can exert a targeted therapeutic effect and relieve apoptosis inhibition. Venetoclax is a new type of BH3 mimetic compound that can target BCL-2 and replace BIM or other regulatory proteins in binding to BCL-2. In this way, venetoclax promotes the release of these BH3-only proteins to activate BAX and BAK, promote their oligomerization and mediate apoptosis [7, 8]. A large number of clinical trials have shown that haematological malignancies are more sensitive to venetoclax than to conventional drugs and that venetoclax has a better therapeutic effect. Currently, the Food and Drug Administration (FDA) has approved venetoclax as monotherapy for the treatment of patients with CLL and small lymphocytic lymphoma (SLL) and in combination with other drugs for the treatment of patients with acute myeloid leukaemia (AML). Although venetoclax has great clinical application value as a new therapeutic drug, studies have shown that many patients still experience relapse in several months to several years after remission [9–11]. Therefore, further research on the specific resistance mechanisms of venetoclax is necessary [12]. In this article, we reviewed and summarized some of the known main mechanisms of resistance to venetoclax to provide a definitive theoretical basis for overcoming clinical venetoclax resistance and further combining it with other drugs (Fig. 1).

### Increased expression of other antiapoptotic proteins

MCL-1 and BCL-XL are antiapoptotic proteins in the BCL-2 family that play an important role in the survival of cancer cells. They are overexpressed in AML, non-Hodgkin's lymphoma (NHL), multiple myeloma (MM) and other cancers [13, 14]. Their mode of action is similar to that of the BCL-2 protein. They bind to BIM to prevent it from binding to BAX/BAK, inhibit the recruitment of BAX/BAK, and ultimately prevent the activation of the apoptotic pathway [8, 15]. ABT-737 is the first-in-class BH3 mimetic compound, and it promotes tumour cell apoptosis through interaction with BCL-2 family proteins. In tumours where the MCL-1 level is low and MCL-1 is absent or inactivated, ABT-737 has a significant effect as monotherapy, but ABT-737 monotherapy is not effective in tumours with high MCL-1 expression. After using RNA interference to downregulate MCL-1 in tumour cells, the sensitivity of the cells to ABT-737 was increased.[16–19] The structure of venetoclax is similar to that of ABT-737; thus, venetoclax resistance may also be related to the level of MCL-1 in tumour cells. Pei et al. retrospectively reviewed 100 newly diagnosed, previously untreated AML patients who received venetoclax combination treatment and found that primary AML cells with a monocyte phenotype were more likely to develop drug resistance than less differentiated AML cells [20]. Analysis of the expression of apoptotic genes showed that the expression rate of BCL-2 in primary AML cells with a monocyte phenotype was decreased, while that of MCL-1 was increased [21]. Moreover, Romain et al. found that the MCL-1 protein content in the venetoclax-resistant (VEN-R) OCI-Ly1 cell line was higher than that in venetoclax-sensitive OCI-Ly1 cells [22]. In addition, MCL-1 amplification and overexpression were observed in the tissues of patients treated with venetoclax [5]. The use of MCL-1 antagonists increases the sensitivity of cells to venetoclax, proving that MCL-1 plays an important role in venetoclax resistance. A variety of MCL-1 inhibitors have been developed and most of them have entered clinical trials, among which S63845, S64315, VU661013, AZD5991, AMG-176, AMG-397, A-1210477 target the MCL-1 protein for binding to dissociate BIM–MCL-1 complexes. After use in combination with venetoclax, not only do they convert leukaemia cells from resistant to sensitive to venetoclax, they also have a synergistic effect in T-cell acute lymphoblastic leukemia (ALL) and diffuse large B-cell lymphoma(DLBCL), recurrent MCL [23–26]. In addition to MCL-1 expression, BCL-XL expression is also increased in VEN-R cells [27–29]. The BCL-XL antagonist A-1155463 can also reverse resistance to venetoclax [30]. Additional studies have shown that the affinity of BIM and other antiapoptotic proteins and thus their importance in venetoclax resistance in CLL follow the order BCL-2 > BCL-XL > MCL-1 > BFL-1. Therefore, BCL-XL plays a greater role than MCL-1 in the drug resistance of tumour cells [27, 31–33]. Kallesh et al. found that for naïve Riva cells, the level of BCL-XL in Riva cells with acquired resistance to venetoclax was increased. However, the expression of MCL-1 did not differ greatly in these two cell lines [34].

The antiapoptotic proteins MCL-1 and BCL-XL confer resistance to venetoclax, and their expression is regulated by related signalling pathways in the cell. The PI3K/AKT/mTOR signalling pathway is an important signalling pathway that regulates cell proliferation, apoptosis and autophagy [35, 36]. AKT can promote the transcription, translation and posttranslational regulation of BCL-2 family proteins by regulating mTOR, GSK3, FOXO and NF-κB [28]. Indeed, the activity of AKT in the VEN-R OCI-Ly1 cell line and the VEN-R SU-DHL-6 cell line was increased to a certain extent compared to that in the corresponding control group. Cells receive signals by interacting with microenvironmental components. Agonists in the microenvironment, such as interleukin-10, CD40L and unmethylated DNA, can stimulate cells through the PI3K/AKT/mTOR signalling pathway to activate NF-κB signalling and promote the expression of MCL-1 and BCL-XL [7, 31, 37]. The effects of inhibitors targeting different components in the pathway also indirectly support this viewpoint. For example, the NF-κB signalling inhibitor BMS345541 can block the expression of MCL-1 and BCL-XL. After treatment with the PI3K, AKT or mTOR pathway inhibitors NVP-BEZ235 and GS-1101, cells resistant to venetoclax become sensitive. The PI3Kδ inhibitor GS-1101 can antagonize the activity of AKT, reduce the expression of antiapoptotic proteins and sensitize VEN-R cells [28, 31].

### Genomic instability

#### BCL-2 mutations

Mutations in drug binding sites are a common mechanism by which tumour cells resist treatment. Many types of gene mutations have indeed been found in the laboratory and case studies of drug resistance to venetoclax; these mutations can inhibit apoptosis by reducing the affinity of the drug and its binding site or affecting the function of proapoptotic proteins [38]. Genetic testing was performed on 15 CLL patients who received venetoclax treatment and progressed. A new BCL-2 mutation, Gly101Val, was found in 7 patients [39]. This new type of mutation decreases the affinity of venetoclax for BCL-2 180-fold [40]. Further studies based on the molecular structure showed that the Gly101Val mutation is located in the BCL-2 α2 helix stacking against the α5 helix and is in the BCL-2 BH3 motif. In this mutant, the valine substituted for glycine is the only amino acid with a prominent large methyl group near the helical backbone of BCL-2, which can provide a stable extension of the helical structure and allow the helix to shift [40]. Therefore, the mutation in this region causes BCL-2 to retain the ability to bind to the BH3 motifs in the regulatory protein, affects the binding of venetoclax to its target site in BCL-2, and significantly reduces the affinity between the two, resulting in resistance to venetoclax. This mutation also prevents the regulatory protein from being released and binding to BAX/BAK, leading to inhibition of apoptosis [41].

With a mechanism similar to that of the BCL-2 Gly101Val mutation, the BCL-2 Phe104Ile mutation was found in a relapsed/refractory FL patient with decreased sensitivity to venetoclax. In this mutation, the phenylalanine at amino acid position 104 in the BCL-2 protein is replaced by isoleucine, which can decrease the affinity of venetoclax to BCL-2 to decrease approximately 40-fold [42]. In addition, two missense mutations F101C and F101L in the same codon in the BH3 domain of BCL-2 were detected in a murine human-like MCL cell line resistant to venetoclax. Both of these mutations prevent venetoclax from binding to the BH3 domain and inhibit mitochondrial apoptosis. In addition, the Phe104Cys and Phe104Leu mutations in BCL-2 also reduce the sensitivity of cells to venetoclax through a similar mechanism [39]. By analysing the genotypes of multiple VEN-R CLL patients, Blombery et al. observed recurrent mutations in the Asp103 codon, resulting in the presence of tyrosine, glutamic acid, and valine residues. However, the Asp103 residue in the P4 pocket is of great importance for the hydrogen binding of the azaindole moiety of venetoclax to BCL-2 protein [43]. Tausch et al. also observed the BCL-2 Asp103Tyr mutation and attached great importance to it in CLL patients with venetoclax resistance. Because this mutation, located in the pocket of BH3, it not only reduces the affinity for venetoclax but also greatly impacts the binding of other proteins. Compared with the abovementioned mutations, the BCL-2 Asp103Glu mutation can reduce the sensitivity of cells to venetoclax, but it does not change the sensitivity of cells to navitoclax, another BH3 mimetic compound. This is because this mutation changes the BCL-2 protein to make it more similar to the BCL-XL protein but does not affect the ability of BCL-2 to bind and release regulatory proteins [40]. Patients who are resistant to venetoclax can harbour a single BCL-2 mutation, but in most patients, two or more mutations are present. For example, patients with recurrent CLL acquire the BCL-2 Gly101Val and BCL-2 Asp103Glu mutations during venetoclax treatment, which seriously affects their prognosis [43].

#### BAX loss

The BAX and BAK genes are the executive genes of the cellular mitochondrial apoptosis pathway. The BAX and BAK proteins encoded by these genes are indispensable for the formation of mitochondrial membrane pores during apoptosis. The function of any BH3 mimic compound requires its downstream effectors BAX and BAK [16, 44]. Therefore, venetoclax-mediated apoptosis of cancer cells greatly depends on the mitochondrial membrane pores and the increased permeability of the outer mitochondrial membrane formed by these two proteins. In AML cells lacking the BAX gene, venetoclax largely fails to induce apoptosis, but venetoclax can trigger apoptosis in cells lacking BAK, indicating that BAX is an important mediator of venetoclax-induced apoptosis [45, 46]. Further studies showed that MV4-11 cells lacking BAX are resistant to venetoclax-mediated mitochondrial depolarization, mitochondrial outer membrane pore formation, cytochrome C release, caspase activation and apoptosis. Knockout of the BAX gene can block the venetoclax-dependent mitochondrial apoptosis pathway; thus, cells with BAX knockout also show resistance to venetoclax. Not only the loss of the BAX gene, but also the mutations of the BAX gene affect the sensitivity of cells to venetoclax [47]. 20 different BAX mutations have been detected in bone marrow or blood samples of VEN-R CLL patients, and more than one BAX mutation, including missense and frameshift/nonsense mutations, was detected in multiple patient samples. The location of the mutation is more important than the type of mutation. Mutations in the hydrophobic part of the C-terminal transmembrane domain of BAX affect the key α9-helix that targets BAX to the outer mitochondrial membrane. These mutations not only lead to a change in the number of α9 helices but also remove the critical terminal amino acids [48, 49]. As a result, BAX cannot anchor to mitochondria, thereby blocking venetoclax-induced apoptosis in vivo and in vitro [50, 51].

#### Changes in other cancer-related genes

TP53 is a highly conserved tumour suppressor gene located in the telomeric region of the short arm of chromosome 17. The encoded protein plays an important role in regulating apoptosis, ageing, DNA repair, autophagy and metabolism [52]. In addition, the mutations in TP53 are also related to disease progression, poor drug treatment effects, and poor prognosis in patients with MCL [53, 54], as well as to venetoclax resistance. Zhao et al. explored the genomic profile of MCL patients who progressed on venetoclax. They found that acquisition of BCL-2 mutations was infrequent; instead, acquisition of TP53 alterations played a role in disease progression on venetoclax. The frequency of TP53 alteration was increased > 2-fold in MCL patients before and after treatment with venetoclax [55]. In addition to MCL patients, TP53 mutations occur in AML patients during the acquisition of resistance to venetoclax [45, 56, 57]. A study by Tamilla et al. showed that compared with 282 wild-type AML patient samples, 16 patient samples with deleterious TP53 mutations showed reduced sensitivity to venetoclax. As TP53 is a gene that controls the expression of proapoptotic proteins, its inactivation or mutation decreases the expression of BCL-2 in AML cell lines. The lower the expression of TP53, the lower is the expression of BCL-2 [45]. Because venetoclax exerts an apoptosis-activating effect by binding to BCL-2, when the level of BCL-2 decreases, the sensitivity of cells to venetoclax decreases [45]. In addition to promoting the expression of the BCL-2 protein, TP53 can also change the level of MCL-1. Studies have shown that the total level of mitogen-activated protein kinase (MAPK) in TP53 mutant cells is increased. The MAPK signalling pathway is involved in the regulation of cell proliferation and apoptosis and can increase the expression of MCL-1 protein, thereby increasing the competitive binding of MCL-1 and BCL-2 to venetoclax and thus affecting venetoclax-mediated cell apoptosis [45, 58–61]. Further studies have shown that the expression level of BCL-XL was increased and the levels of PMAIP1 (NOXA), PUMA and BAK were decreased in a TP53 knockout cell line with reduced sensitivity to venetoclax, which caused inhibition of apoptosis. These cells were resistant to venetoclax-mediated apoptosis. Based on the results of a study indicating that one-third of patients with primary refractory AML resistant to venetoclax carry TP53 mutations, some researchers noted that the integrity of the TP53 gene could be checked to determine whether acquisition of venetoclax resistance was possible in patients with initial or remitted AML to facilitate the selection of a better treatment [62].

A study of the genome of CLL before treatment with venetoclax and after acquisition of venetoclax resistance revealed changes in cancer-related genes other than TP53, such as BRAF, CD274, NOTCH1, RB1, and SF3B1. In addition, mutations in BTG1 and homozygous deletions in CDKN2A/B were detected. These changes may be a major cause of resistance to venetoclax in CLL patients [63]. In AML patients, reconstructed existing mutations, such as expansion of FLT3-ITD, are main reasons for the poor therapeutic effect of venetoclax [40, 64, 65]. Zhang et al. even believed that compared to BCL-2 mutations, expansion of FLT3-ITD was the main contributor to venetoclax resistance in AML [64]. DiNardo et al. conducted a study with 81 AML patients who received venetoclax-based therapy and tracked the corresponding dynamic molecular changes. To identify dynamic molecular changes indicative of adaptive resistance to venetoclax, they compared the variant allele frequency of individual mutations at diagnosis, during remission, and at relapse to identify clones expanded at relapse. Their analysis revealed that relapse associated with progressive clonal expansion of FLT3-ITD was a feature in their study cohort. Expansion of FLT3-ITD can induce ligand-independent autophosphorylation and activation of receptors. Through the PI3K/AKT/mTOR and MEK/ERK pathways and various STAT5 downstream signalling events, the MCL-1 protein level is increased while the expression of BAD and BIM is suppressed to inhibit AML cell apoptosis [62, 66]. FLT3 inhibition by HQP1351 synergizes with BCL-2 inhibitor treatment to potentiate cellular apoptosis in FLT3-ITD mutant AML. HQP1351 inhibits the phosphorylation of FLT3 and its downstream signalling molecules, such as ERK1/2, AKT and STAT5, thus downregulating the antiapoptotic proteins MCL-1 and BCL-XL. In these AML cells, the increased cleavage of caspase 3 and poly(ADP-ribose) polymerase (PARP) prove the enhanced apoptosis. This finding also indirectly proves that expansion of FLT3-ITD is indeed involved in the resistance of cells to venetoclax [67].

#### Abnormal oxidative phosphorylation (OXPHOS)

Amino acid metabolism plays an important role in a variety of malignant tumours—for example, leukaemia stem cells (LSCs) cannot upregulate glycolysis and their survival is thus dependent on mitochondrial amino acid metabolism [68–70]. These cells rely on amino acid metabolism to provide energy for their survival. The combination of venetoclax and the hypomethylation agent azacitidine kills LSCs by decreasing amino acid uptake in these cells, resulting in decreased amino acid catabolism, inhibition of the mitochondrial electron transport chain and consequent inhibition of energy metabolism in LSCs. However, Stevens et al. found that LSCs used fatty acid metabolism to obviate the need for amino acid metabolism, which led to a significant reduction in their sensitivity to venetoclax. Analysis of bulk primary AML specimens revealed the link between mutations in RAS family genes and fatty acid metabolism. Carnitine synthesis, fatty acid metabolism, fatty acid extension in mitochondria, and β-oxidation of long-chain fatty acids in cells with RAS mutations were greatly increased, providing energy for the survival and growth of LSCs treated with venetoclax combinations [71–73]. In addition to the shift from amino acid metabolism to fatty acid metabolism, the components of metabolism were altered in drug-resistant AML LSCs compared with the initial AML LSCs; for example, the level of nicotinamide was increased. The increased level of nicotinamide resulted in increased production of nicotinamide adenine dinucleotide (NAD +), an essential coenzyme that is used in various enzymatic reactions and plays indispensable roles in energy metabolism. LSCs rely on increased NAD + to sustain OXPHOS by promoting the flux of amino acids and fatty acids into the tricarboxylic acid (TCA) cycle. Therefore, the inhibition of energy metabolism induced by venetoclax combination therapy is antagonized [74, 75]. Further studies on VEN-R OCI-Ly1 cells and cases of VEN-R CLL showed amplified chromosomal regions, which contained genes encoding the regulatory subunits of AMP-activated protein kinase (AMPK). As a classic energy sensor, AMPK can not only promote cellular respiration to produce ATP but also limit other physiological processes that consume ATP, maintaining the cell in a high-OXPHOS condition for a long time [76]. Activated AMPK can activate the PI3K/AKT signalling pathway [77–79]. Activation of the PI3K/AKT signalling pathway results in the phosphorylation of specific serine residues of the BAD protein, which binds to the 14–3-3 protein and is sequestered in the cytoplasm, preventing its transfer to mitochondria; in addition, the antiapoptotic proteins BCL-XL, BCL-2 and BCL-W dimerize, thereby blocking apoptosis [80–82]. In addition to the overexpression of AMPK itself, the expression level of its downstream target acetyl-CoA carboxylase is increased. After treatment with the AMPK activator A-769662, the cell line exhibited reduced sensitivity to venetoclax, and after treatment with the AMPK inhibitor dorsomorphin, it exhibited increased sensitivity to venetoclax. The abnormol intracellular OXPHOS caused by activation of the AMPK signalling pathway plays an important role in the decreased sensitivity of cells to venetoclax [83]. The increased MCL-1 in VEN-R AML cells also regulates pathways involved in bioenergetics and carbohydrate metabolism, including the TCA cycle, glycolysis and the pentose phosphate pathway, to change the OXPHOS level in the internal environment.[20, 84] Whether through amino acid metabolism or fatty acid metabolism, primary human LSCs rely on OXPHOS for energy, and in this cell population, OXPHOS at least partially depends on BCL-2 family proteins. Therefore, venetoclax, a highly selective BCL-2 inhibitor, displaces regulatory proteins from BCL-2, promoting oligomerization of BAX or BAK at the mitochondrial outer membrane to initiate apoptosis. In addition, studies have shown that venetoclax can affect cell respiration independently of the BCL-2 family. It can change the level of intracellular OXPHOS by changing the morphology of mitochondria and extensively inhibiting the function of the electron transport chain (complexes I, II, IV) [85, 86]. When venetoclax induces apoptosis in AML cells, hydrolysis of the mitochondrial protein optic atrophy 1 (OPA1) in AML cells increases. This protein plays an important role in maintaining mitochondrial cristae. A reduction in its expression leads to a decrease in the number and detrimental alterations in the morphology of mitochondrial cristae, collapse of the mitochondrial membrane potential and, ultimately, mitochondrial dysfunction. However, AML cells resistant to venetoclax can induce the expression of the OPA1 protein, making the mitochondrial cristae tighter and enriched in the metabolism of amino acids, coenzymes, and ATP [87]. More importantly, these metabolic effects of venetoclax have recently been shown to reciprocally regulate its efficacy [88]. Studies have noted that increased levels of OXPHOS and reactive oxygen species (ROS) confer chemotherapeutic resistance on cancer stem cells [89]. Compared with nonresistant cells, a tumour cell line resistant to venetoclax showed a higher maximum oxygen consumption rate and a higher steady-state level of ROS, and the activity of OXPHOS was indeed significantly increased [22]. Sharon et al. used CRISPR–Cas9 to screen VEN-R cell lines and identified genes associated with venetoclax resistance. These genes encode components of the mitochondrial translational machinery, such as mitochondrial ribosomal protein L54 (MRPL54), mitochondrial ribosomal protein L17 (MRPL17) and ribosome binding factor A (RBFA), which can regulate mitochondrial protein synthesis and alter mitochondrial metabolism. Their abnormal expression results in AML cells becoming resistant to venetoclax. On this basis, they found that the combination of venetoclax and tedizolid, a second-generation oxazolidinone-class antibiotic that blocks mitochondrial protein synthesis, can synergistically activate a heightened integrated stress response (ISR) without altering the expression of BCL-2 family proteins. ISR activation blocks glycolysis, inhibits OXPHOS activity, depletes ATP at the metabolic level, and leads to morphological effects of mitochondrial swelling and vacuolization. The efficacy of the combination regimen of venetoclax and tedizolid indirectly proves that mitochondrial ribosomal proteins play a role in the resistance of cells to venetoclax [90].

## Treatment after venetoclax resistance

The imbalance between antiapoptotic proteins and proapoptotic proteins affects the survival of cells, and one of major mechanisms of resistance to venetoclax is the increase antiapoptotic proteins. We have mentioned that the combination of MCL-1 inhibitors, BCL-XL inhibitors and venetoclax is the most direct method to increase the sensitivity of cells to venetoclax. Based on this principle, BTK inhibitor (BTKi) is also a good choice [91, 92]. It can decrease the expression of MCL-1 protein and increase the expression of BIM protein, while it has no effect on BCL-2 protein level. Therefore, venetoclax and BTKis have a synergistic effect, enhancing the dependence of apoptosis on BCL-2 and increasing the affinity for venetoclax. Victor et al. evaluated the prognosis of 23 CLL patients who received a BTKi after stopping venetoclax due to disease progression [93]. Median progression-free survival and median overall survival were 34 and 42 months in these patients, they considered BTKi therapy to be an effective treatment option for VEN-R CLL patients. An international, multicenter, retrospective cohort study also showed that for VEN-R CLL patients, BTKi resulted in high response rates and durable remissions [94]. Ibrutinib plus venetoclax demonstrat promising efficacy and reliable safety in patients with relapsed/refractory MCL [95, 96]. In addition, DLBCL, FL and AML cells are highly sensitive to ibrutinib combined with venetoclax, suggesting that the combination of these two drugs could be an effective treatment modality for these patients [97, 98]. Besides,arsenic trioxide can lead to the phosphorylation of MCL-1 at Ser159 through PI3K/AKT pathway and attenuate MCL-1 phosphorylation at Thr163 through MEK/ERK pathway, triggering MCL-1 destabilisation and degradation. This causes the release of BIM bound to MCL-1, leading to apoptosis in VEN-R cells [99]. The anti-CD20 monoclonal antibodies (rituximab and obinutuzumab) also alter the expression of BCL-2 family proteins through a similar mechanism, reversing tumor cells resistance to venetoclax-induced apoptosis,making patients benefit from the venetoclax and anti-CD20 monoclonal antibodies combination [100–102].

In addition to affecting the balance between antiapoptotic and proapoptotic proteins, some drugs improve prognosis in drug-resistant patients by altering the level of mitochondrial OXPHOS. Patients undergoing treatment with venetoclax + azacitidine showed not only restricted uptake of amino acid, but also disruption of the TCA cycle and inhibition of electron transport chain complex II. [103, 104] This is a very promising treatment option and AML patients receiving this regimen have achieved high overall response rates in clinical trials [105, 106]. What’ s more,venetoclax combined with azacitidine or chemotherapy regimens are effective and safe in patients with refractory or relapsed acute leukemias of ambiguous lineage, not otherwise specified [107]. LSCs not only rely on amino acid metabolism for energy supply, but fatty acid metabolism plays an important role in these VEN-R cells. 8-chloro-adenosine (8-Cl-Ado), a novel nucleoside analog can interfere with fatty acid metabolism by downregulating important proteins involved in fatty acid oxidation, significantly reducing OXPHOS level in the internal environment, in this way to counteract cellular resistance to venetoclax [108]. In addition, acylchlorohydroquinone and tigecycline can also increase the sensitivity of cells to venetoclax by inhibiting mitochondrial OXPHOS or affecting mitochondrial function [109, 110]. Given the crucial role of mitochondria in cell death and metabolism, many mitochondria-targeted drugs are being studied in order to synergize with venetoclax in the treatment of haematological malignancies.

## Conclusion

**Fig. 2:**
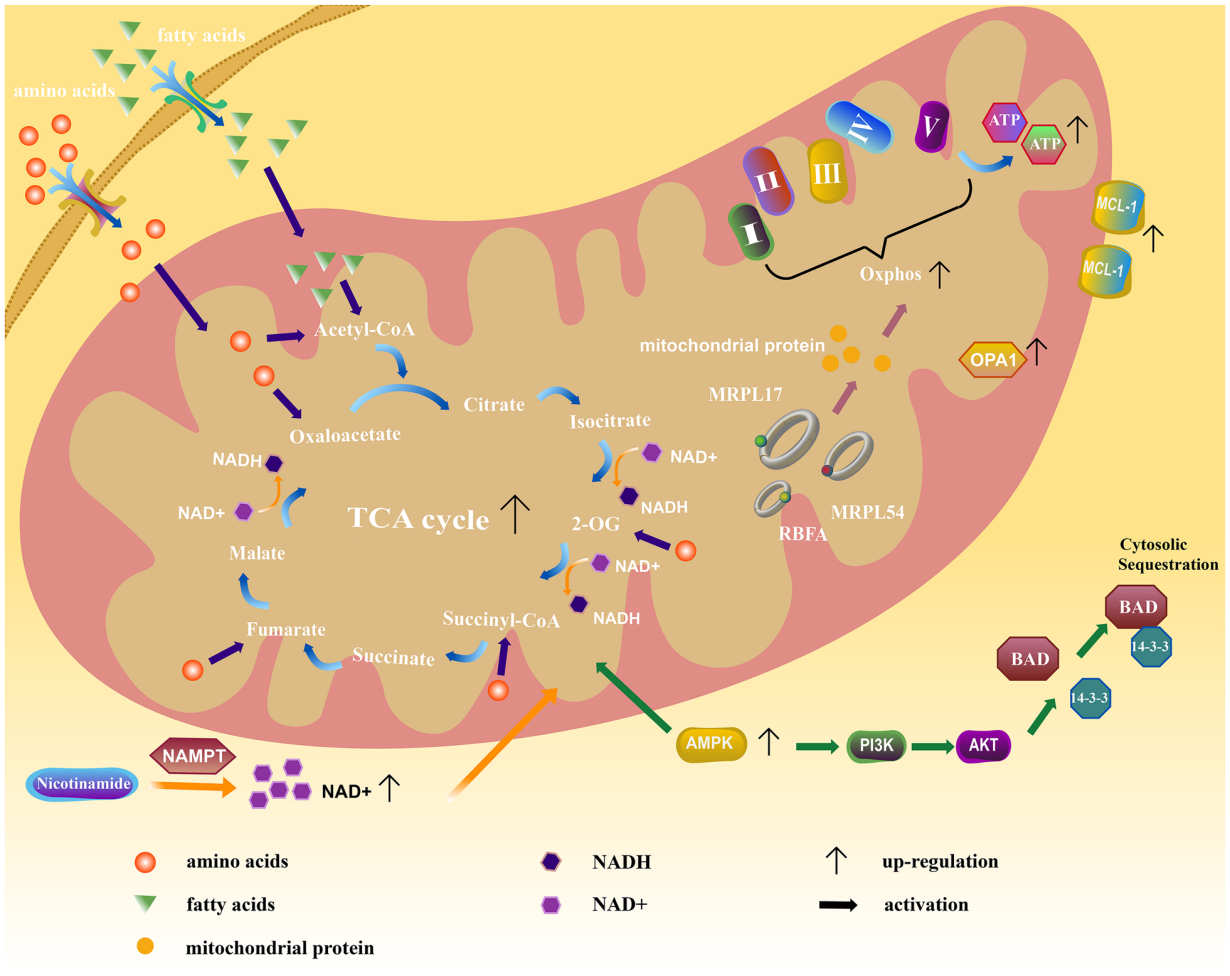
Abnormal oxidative phosphorylation in the mitochondria of VEN-R cells

With the extensive application of venetoclax in haematological malignancies, cases of drug resistance to venetoclax have emerged in current clinical practice. The mechanisms of resistance to venetoclax include increased expression of other antiapoptotic proteins, genetic instability, and abnormal OXPHOS. The antiapoptotic proteins MCL-1 and BCL-XL, as regulatory factors, play a pivotal role in conferring resistance to venetoclax. Although venetoclax binds to BCL-2 and releases BIM, MCL-1 and BCL-XL can directly bind to BIM, which hinders its interaction with BAX/BAK and blocks apoptosis. Genetic instability is the most important reason for decreased sensitivity in patients treated with venetoclax, and mutation of BCL-2 has the most direct effect on this resistance. Although BCL-2 mutations are diverse, their effects are similar. They can reduce the affinity of the binding site for venetoclax and promote the tight binding of BCL-2 to regulatory proteins, making them unable to trigger the apoptotic response. Loss or mutation of the BAX gene also affects the venetoclax-mediated apoptosis process, especially mutations in the hydrophobic part of the C-terminal transmembrane domain, which directly prevents BAX from targeting the outer mitochondrial membrane to form pores. This effect ultimately leads to inhibition of the mitochondrial apoptotic pathway. In addition, mutations in other oncogenes play a regulatory role. In patients with MCL or AML, TP53 mutations induce cellular apoptosis in leukaemia by regulating the expression levels of BCL-2 family proteins through the MAPK signalling pathway. Moreover, in AML patients, reconstructed existing mutations, such as expansion of FLT3-ITD, promote the expression of the antiapoptotic protein MCL-1 and reduce the expression of proapoptotic proteins to interfere with intrinsic apoptosis pathways through the PI3K/AKT signalling pathway. Intrinsic apoptosis depends mainly on mitochondria (Fig. 2). In drug-resistant patients, mitochondria shift from relying on amino acid metabolism to relying on fatty acid metabolism. The level of the important metabolic component nicotinamide increases. These changes antagonize venetoclax-mediated inhibition of energy metabolism, resulting in increased cellular tolerance to venetoclax. Correspondingly, the internal high-OXPHOS environment changes the activity of BCL-2 family proteins and hinders the interactions between venetoclax and these proteins. In addition, venetoclax changes mitochondrial morphology and function alone, forming an internal environment with high metabolism and high OXPHOS levels, which blocks cancer cell apoptosis. Different haematological malignancies have different mechanisms of venetoclax resistance, but all usually involve an interaction of the multiple abovementioned mechanisms. NHL patients not only exhibit expression of antiapoptotic proteins but also show clonal heterogeneity. In addition, abnormalities in mitochondrial OXPHOS are more widespread in AML and CLL patients. We believe that a comprehensive understanding of the venetoclax resistance mechanisms of haematological malignancies will help patients to select appropriate follow-up therapy in clinic and maximize the prognosis of patients. Based on these resistance mechanisms, various clinical trials have been conducted in recent years to explore solutions. We summarize some promising regimens and we consider venetoclax-based combinations to be important therapeutic options for the treatment of haematological malignancies that can be dynamically and individually modified to achieve durable disease remission.

## Data Availability

Data sharing is not applicable to this article as no datasets were generated or analyzed during the current study.

## Abbreviations

FL: Follicular lymphoma
CLL: Chronic lymphocytic leukaemia
MCL: Mantle cell lymphoma
FDA: The Food and Drug Administration
SLL: Small lymphocytic lymphoma
AML: Acute myeloid leukaemia
NHL: Non-Hodgkin's lymphoma
MM: Multiple myeloma
VEN-R: Venetoclax-resistant
ALL: Acute lymphoblastic leukaemia
DLBCL: Diffuse large B-cell lymphoma
MAPK: Mitogen-activated protein kinase
PARP: Poly(ADP-ribose) polymerase
OXPHOS: Oxidative phosphorylation
LSCs: Leukaemia stem cells
NAD +: Nicotinamide adenine dinucleotide
TCA: Tricarboxylic acid
AMPK: AMP-activated protein kinase
OPA1: Optic atrophy 1
ROS: Reactive oxygen species
MRPL54: Mitochondrial ribosomal protein L54,
MRPL17: Mitochondrial ribosomal protein L17
RBFA: Ribosome binding factor A
ISR: Integrated stress response
BTKi: BTK inhibitor
8-Cl-Ado: 8-Chloro-adenosine
